# The ICP-MS Study on the Release of Toxic Trace Elements from the Non-Cereal Flour Matrixes After In Vitro Digestion and Metal Pollution Index Evaluation

**DOI:** 10.3390/foods14081350

**Published:** 2025-04-14

**Authors:** Jiří Nekvapil, Karolína Vilišová, Zdeněk Petřík, Erkan Yalçin, Miroslav Fišera, Robert Gál, Richardos Nikolaos Salek, Martina Mrázková, Martina Bučková, Daniela Sumczynski

**Affiliations:** 1 Department of Food Analysis and Chemistry, Faculty of Technology, Tomas Bata University in Zlín, Vavrečkova 5669, 760 01 Zlín, Czech Republic; nekvapil@utb.cz (J.N.); k_vilisova@utb.cz (K.V.); fisera@utb.cz (M.F.); mmrazkova@utb.cz (M.M.); buckova@utb.cz (M.B.); 2 Department of Health Care and Population Protection, Faculty of Biomedical Engineering, Czech Technical University in Prague, nám. Sítná 3105, 272 01 Kladno, Czech Republic; petrik.zdenek@gmail.com; 3 Department of Food Engineering, Bolu Abant Ízzet Baysal University, Gölköy Campus, 140 30 Bolu, Turkey; yalcin_e@ibu.edu.tr; 4 Department of Food Technology, Faculty of Technology, Tomas Bata University in Zlín, Vavrečkova 5669, 760 01 Zlín, Czech Republic; gal@utb.cz (R.G.); rsalek@utb.cz (R.N.S.)

**Keywords:** pumpkin flour, flax seed flour, milk thistle flour, banana flour, grape seed flour, ICP-MS, toxic trace element, metal pollution index, in vitro digestibility

## Abstract

Detailed research analysis of the contents of eight toxic trace elements in non-cereal flours was conducted using inductively coupled plasma mass spectrometry, and the release of elements from the flour matrixes after in vitro digestion was investigated. It also examines dietary intake and evaluates the metal pollution index. The highest digestibility value was measured with banana flour (92.6%), while grape seed flour was the least digestible, only 44%. The most abundant element was Al, followed by Ni, which was present (except banana flour) at concentrations of more than twice that found in food generally. The flax and milk thistle seed flours showed two orders of magnitude higher amounts of Cd than those measured in other flours. When consuming a 100 g portion of non-cereal flours, a consumer weighing 60 kg is exposed to the highest dietary exposures to Al and Ni (in the order of µg/kg bw); the exposures for the intake of Cd, Sn, Hg, As, Ag, and Pb are of the order of ng/kg bw. Grape seed flour was assessed as a significant contributor to the provisional tolerable weekly intake (PTWI) value of Al (16%); in addition, significant contributions of banana, pumpkin, grape, and milk thistle flours to the PTWI value of Hg, ranging from 15 to 22%, were determined. Furthermore, the contributions of milk thistle and flax seed flours to the provisional tolerable monthly intake (PTMI) value of Cd were also recognized as significant (specifically, 26 and 49%, respectively). The contributions of milk thistle, flax seed, and pumpkin seed flour to tolerable daily intake for Ni were estimated between 19 and 57%. The margin of exposure values for developmental neurotoxicity, nephrotoxicity, and cardiovascular effects obtained for the intake of Pb were considered safe. During the digestion process, the toxic elements that were the most retained in the matrices of grape and pumpkin seed flour were easily released from the banana flour. The retention factor, which was above 50% for Hg in the grape seed flour, was examined as the highest. All toxic trace elements, which were found to still be part of the undigested portion of the flours, could theoretically pass into the large intestine. In the future, more research is needed to clarify the possible carcinogenesis effect of toxic trace elements in the colon.

## 1. Introduction

Toxic trace elements are potentially abundant chemical compounds in the environment due to air, water, or soil contamination. They are mainly sourced from fossil fuel combustion, mining, smelting, electroplating, dyes and pigments, agricultural treatments, and plastic and metallic industries [1]. Toxic trace elements involve both non-metals, such as As, and metals, comprising Cd, Pb, Hg, Al, Ag, Sn, and Ni, which are capable of inducing undesirable effects in living organisms when they exist in high concentrations [1]. Large amounts of toxic elements can reach the human body through the cultivation of plants and crops from contaminated soil, subsequently accumulating in vital organs (such as the liver, brain, kidney, and heart), leading to problems in their normal functioning and blocking vital activity [1]. These toxic trace elements were initially the focus of public health policies as they pose a risk to human health. Therefore, they have been included in legislation and are now being monitored in foods [2]. Plant-derived foods account for at least 70% of Cd uptake in humans, and cereals are the primary source of Cd [3]. Sarwar [4] indicated that Cd compounds are more soluble than other heavy metals, so plants easily take them up and they accumulate in different parts of edible plants. The elemental As is found naturally in all soils and its toxicity is related to solubility, which is affected by pH and redox variations. Rice and rice-based food products constitute the primary sources of human exposure to As [5]. Al was detected at higher concentrations in the food waste study of Kuppusamy [6]; they indicated that plants are just exposed to Al when the soil pH is lower than pH 5.5, and it was explained that this would be dangerous for plant growth [6,7]. Ag is released into the environment from industrial wastes and emissions and is available mainly in the sludge of wastewater treatment plants and surface waters. The exposure of humans to Sn is essentially of dietary origin, coming from the consumption of foods stored in unlacquered tin cans, and may account for 98% of the total ingested Sn [8]. Elementary Ni plays an essential role in the optimal growth of plants because of its structural component of enzymes that catalyze nitrogen fixation. Thus, nitrogen-fixing plants, e.g., soybeans, cereals, and alfalfa, have a high level of Ni administered into the human body. Ni contamination may also occur at the foods’ production, processing, or packaging stages [2].

The use of innovative flours, such as those obtained from agro-industrial byproducts, could not only provide the necessary nutrients for the development of this type of foods increasing their nutritional value [9] but also can be potentially a source of toxic substances. Fruits, vegetables, oilseeds, and beverages are the major food products contributing to food waste, representing serious environmental problems. Besides essential elements, food waste is expected to contain traces of toxic elements that may be detrimental to human health or may exceed environmental permissible limits [6,9]. The peels, seeds, rinds, skin, pulp, or pomace of foods mainly accumulate toxic elements [6]. The food industry also produces many byproducts rich in valuable substances applied as ingredients in functional foods [10]. Banana peel flour, pumpkin seed flour, and grape seed flour are significant food waste, and milk thistle flour and flax seed flour are functional food ingredients utilized in the food industry.

The banana peel waste from industrial processes represents approximately 35–40% of fresh bananas, and about 40 million tons of peel are produced yearly [11,12]. Banana peels have a higher nutrient content than pulp, including protein, fat, dietary fiber, and other essential macro-minerals such as K, Ca, P, and Mg, micro-minerals (Fe and Zn), trace elements (Cu, Mn, B), and vitamin C [13,14,15]. Banana peels contained the following ranges of chemical compounds: 11.9–25.2% ash, 2.41–15.1% protein, 1.76–10.2% fat, 11.1–14.4% dietary fiber, and 25.9–57.1% carbohydrate. Flour is known to treat intestinal lesions, reduce diarrhea, ulcerative colitis, nephritis, and gout, prevent cardiovascular disease, hypertension, and diabetes, and acts as a source of polyphenolic antioxidants [12,14]. Incorporating banana peel into baked goods, such as biscuits, cookies, cakes, and doughnuts, has gained attention due to its potential to enhance flavor, texture, and nutritional value [13].

Pumpkin agro-industry waste, particularly seeds, peels, and pomace, has high levels of carotenoids, dietary fiber, and polyphenols. Pumpkin seeds include significant amounts of antioxidants and crude oil and appreciable amounts of K, Na, Ca, Mg, P, and Fe with trace concentrations of Zn, Mn, and Cu [16,17]. The average crude protein, dietary fiber, crude oil, and carbohydrate content of pumpkin seeds is around 23.7–35.3, 14.2–18.3, 36.6, and 6.02–18.0%, respectively [17]. The amino acids and ω-6 and ω-3 fatty acids of pumpkin seeds play an important role in hormonal balance, brain function, and skin health. By improving fat metabolism, the antioxidant content of pumpkin extract prevents atherosclerosis, heart disease, and high blood pressure [18]. Seeds are also used as a part of functional foods (for example, in salads, baked goods, and snacks) or medicines (e.g., to treat enterozoa) [17,18].

Grape pomace, which is a waste of winemaking, is processed into crude oil via cold processing, and the remaining grape pericarp and seeds are milled into grape seed flour, which can be used as a food ingredient in bakery or pastry products [9,19,20,21]. Grape seeds account for 2–5% of the total weight of grapes and constitute up to 50% of the solid waste in the wine industry [9]. From a nutritional point of view, this by-product consists of dietary fiber, oils, proteins, polyphenols (flavonols and resveratrol), vitamin E, and other important substances, such as minerals, sugars, and organic acids [10,19,21,22]. In some grapes, Ca, K, Mg, Na, and P were considered to be the main minerals contained in the grape seeds, followed by Al, B, Co, Mo, Cr, Fe, Mn, Na, S, Se, and Zn. The structural components of grape seed and skin have been shown to promote healthy functional activities, such as the lowering of LDL cholesterol [23].

Defatted milk thistle seeds are a good source of protein (20.4%), crude oil (11.7%), carbohydrates (38.2%), crude dietary fiber (27.2%), etc. Regarding defatted milk thistle seed flour, Apostol [24] presented a high content of essential elements, such as Ca (9.12 mg/g), K (0.79 mg/g), Mg (4.33 mg/g), Fe (0.81 mg/g), Zn (73.8 µg/g), and Cu (26.9 µg/g). Extracts of thistle seeds containing silymarin are very often used as medical remedies for liver disease, such as cirrhosis, and to prevent liver cancer due to their anticarcinogenic action [24,25]. Biscuit products are one of the most profitable products in retailing, and they are becoming very popular in terms of their medicinal benefits [25].

Filipović [26] reported that flax seed flour includes Ca (18.2 mg/g), Zn (395 µg/g), Cu (119 µg/g), Mn (21.4 mg/g), and Fe (1.09 mg/g). Flax meal is rich in Ca, which, together with the high content of α-linolenic and oleic acids, promotes more significant absorption of this mineral in the intestine and increases its deposition in the bones [27,28]. In addition, flax seed is rich in anti-inflammatory effects and bioactive substances that promote intestinal health, including fermentable dietary fiber (20–30%), which plays an important role in reducing cardiovascular disease, is anti-carcinogenic, and improves brain development in infants [29]. Phenolic antioxidants can alter cell growth and behavior and have medical effects such as lowering blood pressure, preventing diabetes, and protecting neurons [28].

Kuppusamy [6] reported that most of the food wastes studied in their investigation contained Al, as well as Pb, Cd, and As. The skin of pineapple has been found to have Al (590 µg/g), As (0.2 µg/g), and Pb (6.4 µg/g). Apple pomace had the highest concentrations of As (0.2 µg/g), and olive pomace had a high amount of Cd of 2.9 µg/g. In addition, the total toxic element content of pineapple skin (597 µg/g) was higher than that of tea leaves (568 µg/g). In particular, tea leaves were a rich source of Ni (5.0 µg/g). Despite these results, banana peel, plum pomace, and pistachio shell had a toxic element content below the human intake limits [6]; furthermore, the Al content in banana peel was 1.1 µg/g on dry weight, and the As, Cd, Pb, and Ni contents were below the detection limit.

In relation to non-cereal flours, such as banana peel, pumpkin seed, grape seed, milk thistle, and flax seed flours, the profile of toxic trace elements, their daily dietary intake, and exposures, including their metal pollution index, have not been reported, in detail, in the literature until now. The primary objective of our study was to investigate the content of Al, Ni, As, Ag, Cd, Sn, Hg, and Pb as the main toxic trace elements in native and undigested portions of these food waste and food ingredients using the ICP-MS method. A two-stage simulation of the digestion process in the stomach and small intestine was applied under in vitro conditions to prepare the undigested portion and to determine the digestibility values of non-cereal flours. Furthermore, this study was designed to estimate the contributions of non-cereal flours to provisional tolerable weekly intake (PTWI), provisional tolerable monthly intake (PTMI), tolerable daily intake (TDI), and oral reference dose (RfD) for individual toxic trace elements, based on the evaluation of daily dietary intake (DI) and exposure (DE_bw_) at 60, 80, and 100 kg adult body weights. Additionally, our evaluation included the retention factor (RF) and metal pollution index (MPI) as risk-assessment values for human health.

## 2. Materials and Methods

### 2.1. Chemicals and Reagents

Pepsin with an activity of 2000 FIP-U/g and a pancreatin enzyme complex with activities of 7500 FIP-U/g for amylase, 350 FIP-U/g for protease, and 6000 FIP-U/g for lipase from a porcine pancreas were purchased from Carl Roth and Merck (Karlsruhe and Darmstadt, Germany). ICP-MS 8-standards (Al, Mn, Co, Ni, Cu, Zn, Sn, and Ag), ICP-MS 7-standards (Cr, Fe, As, Se, Cd, Hg, and Pb), and ICP-MS internal Rh standard were obtained from Analytika (Prague, Czech Republic). Tune 7 solution was purchased from Analytika (Prague, Czech Republic). Gases (Ar and He) were supplied from Linde Gas (Zlín, Czech Republic), and ultrapure water was prepared using a Purelab Classic Elga water system (Labwater, London, UK). Certified reference materials of NIST rice flour 1568b and lichen were purchased from Analytika (Prague, Czech Republic) and the International Atomic Energy Agency (Vienna, Austria), respectively.

### 2.2. Sample Collection

The commercial material studied consisted of five non-cereal gluten-free flours: banana peel flour (dry and milled banana peel, *Musa* spp.), pumpkin seed flour (dry and milled seeds, *Cucurbitaceae*), grape seed flour (dry and milled seeds from grapes, *Vitis vinifera*), milk thistle seed flour (dry and milled seeds, *Silybum marianum*), and flax seed flour (dry and milled brown type of linen seeds, *Linum usitatissimum*) (Adveni Medical, Mělčany, Czech Republic). The samples were collected in 2023 with an expiration date of 2024. They were kept in the original 0.25 kg packs out of sunlight in an air-conditioned laboratory (23 ± 2 °C) for a period of less than one month prior to analysis.

### 2.3. Determination of Digestibility Value

**Figure 1:**
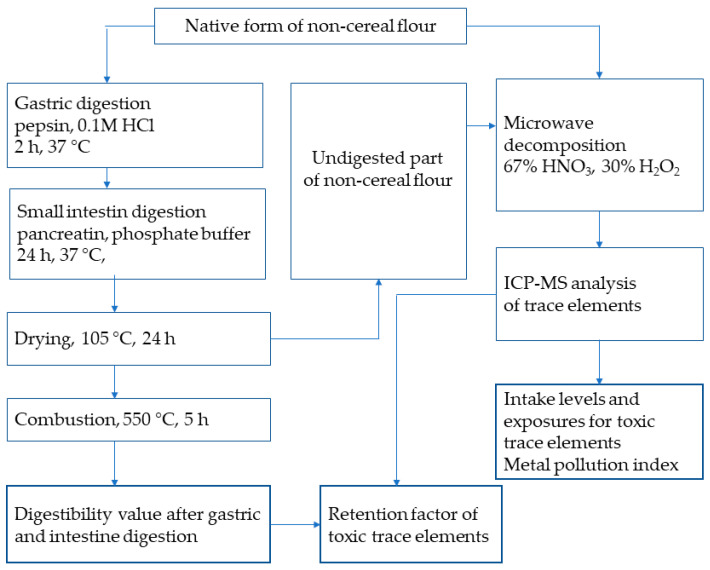
Experiment procedure.

The gastric and intestinal phases as part of the in vitro digestion process were performed according to [30] (Figure 1).

The sample was subjected to hydrolysis with pepsin and then to a mixture of pancreatic enzymes in a Daisy incubator (Ankom Technology, Macedon, NY, USA). The determination of dry matter and ash content for calculating the in vitro digestibility value (%) was carried out according to [31]. First, the non-cereal flours (0.25−0.30 g) were weighed into F57 bags (Ankom Technology, Macedon, NY, USA) and, consequently, sealed with an impulse sealer (KF-200H, Penta Servis, Holice, Czech Republic).

The polyethylene incubation flask was filled with 1.7 L of 0.1 M HCl (pH 2.5) containing pepsin (0.63 g) to simulate gastric processes. The sealed samples were digested for 2 h at 37 °C and then rinsed with ultrapure water (18.2 MΩcm, Purelab Classic Elga equipment, LabWater, London, UK). To simulate conditions in the small intestine, 1.7 L of phosphate buffer (pH 7.45) was dissolved in ultrapure water and a mixture of pancreatin enzymes (3.0 g) was added to the incubation tube.

After the incubation, the samples were rinsed multiple times with ultrapure water, dried at 105 °C for 24 h (Venticell oven, BMT Medical Technology, Brno, Czech Republic), and weighed. Finally, the sealed samples were combusted in a muffle furnace (LM112.10, Veb Elektro, Berlin, Germany) at 550 °C for 5 h, cooled in an exicator, and weighed. The in vitro digestibility value was calculated according to [30].

To obtain undigested residues of the non-cereal flours, the digestibility process was terminated by drying the samples at 105 °C for 24 h. Subsequently, the solid undigested residues of the flours were decomposed using 67% HNO_3_ and 30% H_2_O_2_ (see Figure 1).

### 2.4. ICP-MS Analysis

Toxic trace elements (Al, Ni, Sn, Cd, Pb, As, Ag, and Hg) were measured in the native form of the flours and in the undigested part of the flours to assess their concentrations and levels of dietary intake and retention after digestion and to evaluate the metal pollution index. A Milestone Ethos One microwave decomposition oven (Milestone Laboratory Systems, Sorisole, Italy) was used to mineralize the flour samples. To decompose the sample, about 0.2 g of the samples was weighted, transferred inside polytetrafluoroethylene (PTFE) vessels, and 7 mL of 67% HNO_3_ and 1 mL of 30% H_2_O_2_ (both in analpure quality, Analytika, Prague, Czech Republic) were added. The instrumental conditions used for the microwave digestion were 500 W for 10 min at 150 °C, 1200 W for 15 min at 160 °C, and, finally, 500 W for 15 min at 150 °C. The clear and colorless mineralizate was transferred to PET test tubes and brought to a volume of 25 mL using ultrapure water. From each non-cereal sample, three aliquots were digested, and each sample solution was analyzed in triplicates using ICP-MS [30]. The toxic trace element analysis was performed using a Thermo Scientific iCAP Qc inductively coupled plasma mass spectrometer (Thermo Scientific, Waltham, MA, USA) equipped with a collision cell (QCell) containing He (CCT: collision cell technology; KED: working in kinetic energy discrimination mode) (Table 1) [30].

The daily performance of ICP-MS in terms of sensitivity and background signals was tested using the Tune 7 solution with Ag, Al, Co, Cu, Mn, Ni, Rh, and Zn containing 1 μg/L of each element in 2% HNO_3_. To obtain calibration curves, two sets of standards were prepared to match the concentration ranges in the samples: a high standard series of three elements (^27^Al, ^60^Ni, ^107^Ag) at concentrations of 3–35 μg/L and a low standard series of five elements (^75^As, ^111^Cd, ^118^Sn, ^202^Hg, and ^208^Pb) with concentrations between 0.5 and 2.0 μg/L. As an internal standard, rhodium (^103^Rh) was used at concentrations of 5 μg/L.

The certified reference materials of NIST Rice flour 1568b from the National Institute of Standards and Technology (via Analytica Ltd., Prague, Czech Republic) and the lichen supported by the Atomic Energy Agency (Vienna, Austria) were applied to evaluate the measurement accuracy of the measurement using of recovery as a validation parameter (Table 2).

### 2.5. Dietary Exposure Assessment

Appropriate dietary intake levels for toxic elements were calculated for human body weight (bw) averages of 60, 80, and 100 kg. Since there is no recommendation for the daily intake of non-cereal flours, the daily serving size was set to 100 g for each examined bw. The estimated daily dietary intakes (DI) of toxic trace elements from consuming the non-cereal flours were calculated using Equation (1).

(1) $$DI={CA}_{flour}\times {SS}_{flour}$$

where DI is the daily dietary intake (µg or ng per day), CA_flour_ is the content of analyte in non-cereal flour (µg or ng /g), and SS_flour_ is the serving size of the non-cereal flour (100 g). For the estimate of the daily dietary exposure by body weight (DE_bw_) (Equation (2)), the DI value was divided by the weight of the participants [32,33,34].

(2) DEbw=DIbw

The intake levels of toxic trace elements were also evaluated and compared with the PTWI or PTMI values as suggested by the authors in [35,36,37,38]. The PTWI reference values for Al, Sn, and Hg were established as 2000, 14,000, and 4 µg/kg bw, respectively; similarly, the PTMI reference value of 25 µg/kg bw for Cd was applied. In our study, TDI levels of 13 and 0.3 µg/kg bw were mentioned to evaluate the Ni and As intake levels, respectively [39,40,41]. In the case of intake of Ag, its RfD of 5 µg/kg bw was applied to calculate exposures from consumption of non-cereal flours [42]. To define the risk characterization for Pb, the margin of exposure (MoE) approach was applied (Equation (3)). It was calculated from the ratio of the Benchmark Dose Level (BMDL) to the estimated DE_bw_ value to Pb, considering the respective individual BMDL values for developmental neurotoxicity (BMDL of 0.5 μg/kg bw/day), nephrotoxicity (BMDL of 0.63 μg/kg bw/day), and cardiovascular effects (BMDL of 1.5 μg/kg bw/day), as prescribed in EFSA [43,44].

(3) MoE=BMDLDEbw

### 2.6. The Effect of In Vitro Digestion on the Toxic Element Content of Non-Cereal Flours

The amounts of toxic trace elements still remaining in the undigested portion of the flours after simulating the digestion process were evaluated as the retention factors (RFs), and the results were expressed as a percentage (%). The RF value for each analyte was calculated using Equation (4).

(4) $$RF=\frac{C_{UP}\times (100-Digestibility)}{C_{NP}}$$

where RF is the retention factor of the appropriate analyte in the undigested part of the sample (%), C_UP_ is the concentration of the analyte in the undigested part of the flours (ng/g), the in vitro digestibility value of the flours is expressed in %, and C_NP_ is the analyte concentration in the native form of the flours (ng/g) [30].

### 2.7. Metal Pollution Index Evaluation

To examine total toxic metal concentrations in non-cereal flours and in their undigested parts, the metal pollution index (MPI) was calculated. This risk assessment index was calculated using the geometric mean of the concentrations of toxic metals contained in the flours (Equation (5)) [45].

(5) $$MPI={\left(C_{1}\times C_{2}\times \ldots C_{n}\right)}^{1/2}$$

where MPI is the metal pollution index (-), and C_1_, C_2_, and C_n_ are the concentrations of an individual metal in the native and undigested part of the flour. To evaluate which part of the MPI value is still maintained in the undigested part compared to native flours, the remaining parts of the metal pollution index (RP_MPI_, %) were calculated as follows (Equation (6)):

(6) RPMPI=MPIUP×(100−Digestibility)MPINP

where RP_MPI_ is the remaining part of the MPI index still maintained in the undigested part of the flours (%), MPI_UP_ is the metal pollution index of the undigested part of the flours (-), the in vitro digestibility value of non-cereal flour is presented in %, and MPI_NP_ is the metal pollution index in the native part of the flours (-) [30].

### 2.8. Statistical Analysis

Trace element analyses were performed nine times; the digestibility assay and the dry matter and ash content analyses were repeated three times. The results were expressed as mean ± standard deviation on a dry weight basis and were statistically evaluated using one-way analysis of variance (ANOVA, TriloByte Statistical software, QC expert 3.3. Pardubice, Czech Republic). Subsequently, Tukey’s test was used to identify differences between the mean values; the level of significance was set to 5% (*p* < 0.05).

## 3. Results and Discussion

### 3.1. Toxic Trace Element Content of Non-Cereal Flours

In recent decades, numerous research papers have monitored the content of essential elements in non-cereal flours prepared from seeds and pomace [15,17,19,46]; nevertheless, data regarding toxic trace elements in non-cereal flours are scarce. Therefore, eight toxic trace elements were assessed using ICP-MS in non-cereal flour (banana, pumpkin, grape seed, milk thistle seed, and flax) and their undigested parts. The content of minerals and trace elements in plants and their parts is affected by many factors, for example, variety, state of ripeness, climate, soil condition, fertilization and irrigation, method of cultivation, and, last but not least, method of processing into final products [15].

As can be seen in Table 3, the highest concentration values (5220−27,500 ng/g) were measured in the case of Al determination. The average Al content of fresh banana pomace can vary widely, from 0.05 to 32.8 µg/g [47], while the banana flour used in this study (produced by drying and milling the pulp) contained an Al level of about 4.5 times lower than the previous upper limit. It may be emphasized that the significantly highest Al content was found in grape seed flour (27,500 ng/g), which was three orders of magnitude lower in concentration than was measured in grape pomace [22]. The latter phenomenon can be explained by the fact that only from 2 to 3% of the total amount of Al is accumulated in the seeds; the remaining part is located in the skin and pulp of grape berries [48]. In addition, the amount of Al can vary widely between different cultivars of the same fruit [49]. The main source of Al is the diet, specifically, cereals, cereal-based products, and vegetables [32]. The Agency for Toxic Substances and Disease Registry (ATSDR) includes Al, along with As, Pb, Hg, Cd, and Ni, on its Substance Priority List (SPL) for toxic compounds [50]. So far, the maximum certain level for the occurrence of Al in foodstuff has not been set yet.

Although Ni is not essential for humans, it is studied as a trace element because of its potentially harmful health effects. For the time being, the maximum allowable concentration value for Ni as a contaminant in food has not yet been established. There are many studies in the research literature on the occurrence data of Ni in food [2,49,51]. The highest level of Ni in the human body is taken with drinking water, legumes, nuts, oilseeds, tofu, dark chocolate, and breakfast cereals [2]. In general, Ni has been measured in a variety of foods in average concentrations below 500 ng/g [52], but it can even reach 12–15 µg/g [51,53]. The total concentration of Ni in various food commodities depends on the type of food, growth conditions, especially on environmental conditions, and the raw material processing technology [53]. It is evident that only the banana flour sample met the abovementioned limit. In general, fruit is not assumed to contain high amounts of Ni. For instance, fruit puree can contain between 66 and 1450 ng of Ni per gram of fresh matter [49]. The highest mean concentrations of Ni have been measured in beans, oilseeds, and grains with concentration values of 9800, 5100, and 2300 ng/g, respectively [52,54]. Regarding non-cereal flours measured in our study, the highest Ni value was assessed in milk thistle flour (4430 ng/g) followed by flax seed flour (1810 ng/g) (see Table 3). In the group of pumpkin and flax seeds and raisins, the mean reported concentrations of Ni were 1800, 1200, and 200 ng/g, respectively [52,54]. Our observations, in general, agree with the reported literature data. Furthermore, a 10% addition of pumpkin seed flour to wheat bread was measured to increase the Ni content by 0.5 mg per kg of bread [55].

The As concentration in non-cereal flours was found to be between 6.32 and 14.4 ng/g, whereas the highest value was measured in milk thistle flour (Table 3). In food, most toxic inorganic As species are predominantly in the oxidation state of As^3+^ or As^5+^, present as thio complexes or as oxo anions, arsenite, and arsenate [56]. The inorganic form of As is known to be a genotoxic carcinogen. The main means of exposure to arsenic in the general population is through contaminated food and water intake, where the inorganic form of As is predominantly found in cereals, and the organic form is prevalent in fruit and vegetables [53]. To date, the maximum value for the remaining limit of As in foods has been established only for the inorganic form of this element, which is allowed up to a maximum of 0.30 mg/kg in rice wafers, rice crackers, rice flakes, and popped breakfast rice and 0.25 mg/kg in the case of rice flour [57]. There is no limit for the maximum value of As in non-cereal flours and their raw materials. Rice contains significant As levels with concentrations often between 0.1 and 0.4 mg/kg of dry mass, sometimes considerably similar to nuts; for example, the maximum level of As concentration in fruit matrices was found to be approximately 2.20 mg/kg. In contrast, oilseeds can reach As contents of up to 5.70 mg/kg [58]. Samples of non-cereal flours do not reach these concentrations. For the occurrence of As, there are not enough data available in the literature on its content in non-cereal flours to be reasonably compared with the results obtained in the present work. To compare data, Bertoldi [48] published the As value in grape berries with a mean value of 371 µg/kg on fresh mass, while the As content in grape seeds, depending on geographical location, can range from 0.10 to 0.70 µg/g on dry matter of the sample [20].

Regarding Ag concentrations in non-cereal flours, the highest content (6.61 ng/g) was found in grape seed flour, while very low amounts of Ag were measured in milk thistle (0.23 ng/g) and flax seed (0.53 ng/g) flours. Although research studies based on measurements of Ag values in grape seed flours are scarce, Bertoldi [48] described the accumulation pattern and proportional distribution of Ag in different parts of grapes, which were quite similar. Concerning distribution, Ag has been accumulated mainly in the flesh. This could explain the low Ag concentrations in the grape seeds ground to flour, as the mean value of the Ag content in the pulp of the technologically ripe grapes was up to 1.18 µg/kg [48]. This assumption can be supported by another study [20], where Ag was at concentrations below the microgramme range (concretely, from 0.04 to 0.25 µg/g). For this element, it has to be mentioned that there are not enough data available in the literature on its content in non-cereal flours to be reasonably compared with the data obtained in the present research study. Generally, the amount of Ag absorbed by several plants is related to its content in the soils, especially in plants growing in Ag-mineralized areas. To date, maximum levels for Ag as a contaminant in any food have not been established, although Ag is listed in the SPL for toxic compounds [50].

When analyzed for the presence of Cd, it should be noted that flax and milk thistle flours contained the significantly highest concentration values (247 and 132 ng/g, respectively), which were two orders of magnitude higher than those measured in banana peel, pumpkin seed, and grape seed flours. High levels of Cd pollution in the atmosphere and soil can lead to plant phytotoxicity and consequently result in the transfer of heavy metals from crops to the human diet [53,59]. When dietary intake through contaminated foods is the main route for human heavy metal intake, the European Commission (EC) strictly regulates the allowable contents or maximum permitted concentrations of toxic heavy elements in foodstuffs. The Commission Regulation [57] prescribes maximum Cd concentrations in certain types of cereals (50−180 ng/g), fresh bananas (20 ng/g), fresh berries (30 ng/g), oilseeds (100 ng/g), and flax seeds (500 ng/g). There are no limits for Cd content in appropriate non-cereal flours. Concerning our samples, the flaxseed flour reaches approximately half the maximum value for the Cd content set in the EU regulation. Theoretically, if we compared the Cd content of the milk thistle flour sample with the maximum allowed limit for its content in oilseeds, this sample would not meet this defined value. Given that milk thistle flour is made by milling seeds after the oil is extracted, it would be a good idea, in the future, to check the content of Cd of this raw material or to set its maximum level limit. All other non-cereal flour samples measured in this study contained Cd levels below the values specified for the food categories mentioned above. It seems that the Cd content in banana flour is so low that even a 15% addition of banana flour to a wheat biscuit recipe did not increase the concentration of this element [12]. Regarding gluten-free flours, the Cd content was in the range from 2.60 to 3.00 ng/g [51], which is comparable with the Cd value in grape seed and banana peel flours. The Cd content in non-cereal flours has not yet been sufficiently substantiated. Nevertheless, ground flakes are often used in home conditions to prepare flour. The coarse oat and wheat flakes can contain 16−53 and 19.6−21.9 ng of Cd per 1 g of dry matter sample, respectively, while no cadmium was detected in the rice flakes [60].

The inorganic chemical form of Sn is considered to have a very low toxicity due to its slow absorption rate and solubility in the human digestive tract [35]. Despite the fact that Sn is ordinarily present only in trace amounts in food, high exposure to this metal could result in adverse health effects such as neurodegenerative diseases and gastrointestinal problems [61]. Concerning these statements, the upper regulatory limits for Sn content have been established only for canned food and beverages, which were set at 200 and 100 µg/g, respectively [57]. At first glance (see Table 3), the Sn content in the non-cereal flours was significantly lower than these limits. To date, almost no attention has been paid to monitoring the Sn content in non-cereal flours, so comparing the measured data with other studies is difficult. To compare at least in similar food matrices, the Sn content measured in cereals, fresh fruits, and seeds reached the following concentrations: 2470, 353, and 845 ng/g, respectively [61]. Our results can also be compared with the study published by Millour [62], where Sn contents in breakfast cereals, oilseeds, and fruits (determined in the concentration ranges 2−7, 13−29, and 2−39 ng/g, respectively) are of the same order of magnitude as in non-cereal flours.

Hg is hazardous and is classified as a trace element that is not essential for humans. The toxicity of Hg depends on its chemical form [44]. The most toxic is organically bound Hg (e.g., methylmercury, found mainly in fish, and meat products, which is more easily bio-accumulated during food consumption), while the divalent Hg ion bound to inorganic complexes dominates in the plant matrix and is less toxic [63,64]. The toxicity of Hg is most often directed towards the kidneys, followed by damage to the human immune and reproductive systems [44,63]. Because of the neurotoxicity of Hg compounds, their content in fishery and meat products and food supplements is under the control of legislation in the EU [57]. Toxic limits for Hg content in foods of plant origin have not yet been established. In the target of our measurement (Table 3), the Hg values were found in the range of 17.4 and 75.1 ng/g. Data on the Hg content in non-cereal flours are not sufficiently available. However, for comparison, the results of this study can be discussed with the conclusions of the Scientific Opinion of the EFSA (The European Food Safety Authority). The grain-based products, oilseeds, and fruits consumed in the EU were stated to contain up to 3.10, 3.30, and 2.10 ng/g of Hg, respectively [63]. Bertoldi [44] measured even lower Hg concentrations in wine grapes, namely, 0.26 ng/g of fresh matter, with the highest occurrence recorded in the pulp, and no Hg content was detected in the seeds. Furthermore, gluten-free flours can contain 0.50−1.00 ng of Hg per gram [51], cereal grains and fruits 4.40 and 1.90 ng/g [44], and wheat grains 4.00 ng/g [65]. It is evident that the samples analyzed in this study were found to have a Hg content of the order of tens of nanograms.

Pb is generally considered to be a potentially toxic trace element that has been associated with health problems such as neurotoxicity and nephrotoxicity, anemia, and gastrointestinal symptoms [44,66]. Its intake occurs mainly through consuming contaminated food; the other exposure pathways represent drinking water and air [66]. Although Pb concentrations in commonly consumed foods are generally low, concerning Pb toxicity and its accumulation in the liver and kidney, maximum allowable concentration values have been introduced in the EU for a specified food commodity. For instance, the Commission Regulation [57] determines the maximum level of Pb for fruit (100–200 ng/g) and cereals (200 ng/g). No limit has been set on the concentration of Pb in non-cereal flours. As can be seen from the measurement results displayed in Table 3, none of our samples exceeded the limits mentioned above. With regard to the fact that the content of toxic elements in non-cereal flours has not yet been described in the literature, it is challenging to discuss the measured data. For example, wheat grains can contain a wide range of Pb concentrations (from 22 to 270 ng/g) [59], similar to gluten-free flours (1.30–30.0 ng/g); even higher Pb values can be found in grape seeds (up to 362 ng/g) [21]. The results processed by [21] could hypothetically explain the highest concentration of Pb in grape seed flour (83.4 ng/g).

### 3.2. Dietary Exposure to Toxic Trace Elements from Non-Cereal Flours

Regarding non-cereal flours, plants can uptake toxic elements from soils, fertilizer, irrigation water, and atmospheric dust [65]. When considering different pollutant compounds, trace elements in the toxic metal group represent an exceptional risk due to their persistence and toxicity. Potentially toxic elements cause various cancers, neurological problems, thrombotic diseases, etc. The intake of Al, Ni, As, Ag, Cd, Sn, Hg, and Pb occurs mainly through the consumption of contaminated foodstuffs [65,66]. In particular, As, Cd, Pb, and Hg are listed in the top ten components of the SPL created by the ATSDR based on their occurrence, toxicity, and potential harmful effects on health [50]. Thus, not only the contamination of waste by-products (used for producing non-cereal flours) should be of public interest but also their contributions to the dietary intake and exposure to toxic elements. Estimated daily dietary intakes (DIs) and dietary exposures (DE_bw_) by body weight for Al, Cd, Sn, and Hg from consumption of non-cereal flours are presented in Table 4.

**Figure 2:**
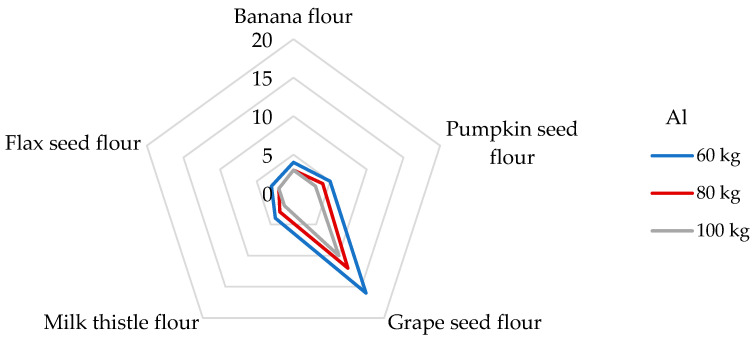
Dietary intake exposures (%) of Al from non-cereal flours with respect to body weight (kg).

The DI values for Al after serving 100 g of non-cereal flours ranged from 522 to 2750 µg/day. The highest DI value was evaluated in grape seed flour, and the lowest value was recorded in flax seed flour (Table 4). When consuming flax seed and grape seed flours, a person weighing 60 kg is exposed to DE_bw_ values of 8.70 and 45.8 µg/kg bw, respectively. A large amount of Al is incorporated into the minerals of aluminum silicate in the soil, and the soluble forms of Al are capable of influencing biological systems. Exposure to Al is related to anemia-type symptoms, bone disease, and dialysis encephalopathy; in addition, overexposure to Al can increase the risk of Alzheimer’s dementia, amyotrophic lateral sclerosis, and Parkinson’s disease [7,32,67]. In 2008, EFSA stated that the mean dietary exposure from water and food exhibited significant variations between different countries, ranging from 1.60 to 13.0 mg of Al per day, which corresponds to from 0.20 to 1.50 mg/kg of body weight per week in a 60 kg adult [32]. Cereal and cereal products appear to be the main contributors to dietary exposure to Al, followed by vegetables and fruits, which are very often processed ingredients for children’s snacks [32,49]. Different results were published in the study by Filippini [61], where the average total dietary intake of Al was 4.1 mg/day, corresponding to an intake of 58.2 µg/kg per day for a person weighing 60 kg. Instead, as the main contributors to the Al intake, vegetables were evaluated, followed by coffee and tea, and, finally, cereal products. The study mentioned above determined the Al intake from 100 g of cereals 347 µg/day, which is even eight times less than in the case of the consumption of grape seed flour. It should be noted that the non-cereal flours analyzed in our study will probably exceed the Al intake from cereals. Interestingly, a high amount of Al was observed in plant raw materials associated with the presence of Si. A non-negligible role of Si is in the prevention of the re-absorption of Al in the kidneys, thus increasing the urinary excretion of Al [49]. For this reason, it would be appropriate to determine Si concentrations for plant materials that show higher DI values for Al in the future. Concerning the potential health risk of consumption of Al, the JECFA (Joint FAO/WHO Committee on Food Additives) set the PTWI value for Al at 2 mg/kg bw [37]. As shown in Figure 2, dietary exposure levels of non-cereal flours to the PTWI of Al varied from 3 to 16% for adults weighing 60 kg, whereas the highest contribution was found for the grape seed flour. The lowest contribution to PTWI was calculated for flax seed flour, where a 100 g portion contributed 3 and 2% of PTWI for a participant weighing 60 and 100 kg, respectively. For example, a higher significant contribution to the value of PTWI for Al (up to 25%) was estimated from a 100 g portion of rice for an adult weighing 70 kg [68].

**Figure 3:**
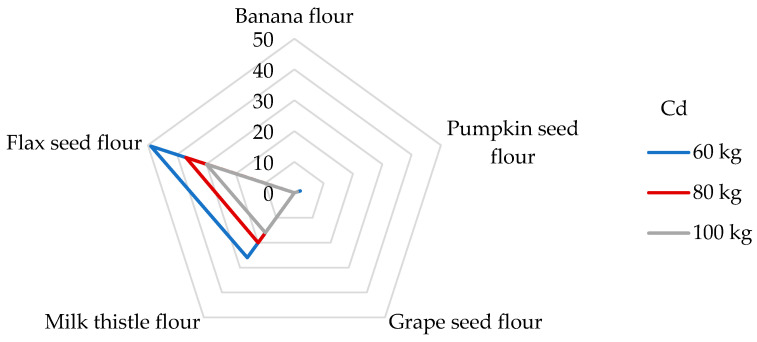
Dietary intake exposures (%) of Cd from non-cereal flours with respect to body weight (kg).

Although Cd absorption from the diet is relatively low (3–5%), Cd is known to cause bone demineralization and renal dysfunction and may be retained in the lungs, kidneys, and liver [65,69]. Because Cd displaces Zn from many metallo-enzymes, chronic exposure to Cd leads to Cd-induced Zn deficiency [3]. The International Agency for Research on Cancer (IARC) has classified Cd as a human carcinogen [53], and it occupies the seventh position on the ATSDR’s Substance Priority List [50]. The main food categories that contribute to the daily intake of Cd include grain products (27%) and vegetables (16%) [70]. As can be seen in our study (Table 4), the highest estimated DI values were observed in flax seed (24.7 µg/day) and milk thistle seed flours (13.2 µg/day). If the daily intake of Cd in the USA from food is around 18.9 µg/day [53], then the consumption of 100 g of flax seed flour exceeds this value. In most countries, the average daily exposure of Cd in food is in the range of 0.1–0.4 µg/kg bw [53]. DE_bw_ values for flax seed and milk thistle flours were evaluated at 247–412 and 132–220 ng/kg bw, consistent with the published data. Regarding the EU datasheets, the total daily dietary exposure of Cd was estimated to be in the range of 271 and 430 ng/kg bw for non-vegetarians and up to 770 ng/kg bw for vegetarians [53,71]. All samples of non-cereal flours meet these limits, but it should be noted that other foods consumed per day may also contribute to the total Cd intake instead of non-cereal flours. The estimated exposure to Cd was in the same range as that observed in the studies by Kafouris [44] and Wong [72]. Different results were achieved, where the daily Cd intake when consuming a 100 g portion of corn kernels was 8.64 ng/kg bw [65], which is eleven times lower than when consuming a 100 g portion of grape seed flour. In 2013, the JECFA Expert Committee on Food Additives established the PTMI of 25 μg Cd per kg bw [38], which corresponds to a previously suggested daily exposure of 830 ng/kg bw [73]. It is evident (Figure 3) that the contributions of milk thistle and flax seed flours to the PTMI values for Cd are significant, specifically, 26 and 49% for people weighing 60 kg, respectively. Banana, pumpkin, and grape seed flours (Figure 3) were not significant contributors to the PTMI value of Cd and vice versa. For example, a 100 g portion of rice provides between 7 and 10% of the PTMI for Cd [68] for a person weighing 65 kg; similarly, in the study determining Cd in wheat grains, a contribution of 13% was found for wheat-based products [60]. Similar results were obtained for gluten-free products for celiac patients [51]. Milk thistle and flax seed flours appear to be considered a strong potential source of Cd intake concerning their daily portion size.

Sn is relevant in assessing adverse health effects regarding different chemical forms. Exposure to organic chemical forms, such as trimethyltin and triethyltin, is often associated with metabolic, gastrointestinal, and neurological effects; adversely, inorganic forms of Sn cause mainly gastrointestinal problems. The primary source of exposure to Sn in the diet is packaging materials made from tin plates, as they significantly increase exposure to this trace element [33,61]. The daily value of the dietary intake of Sn from the consumption of non-cereal flours ranges from 507 to 1470 ng/day, which corresponds to a dietary exposure of from 8.45 to 24.5 ng/kg bw for the participant weighted 60 kg (Table 4). When comparing the data with the study presented by Filippini [61], it was found that the mean total daily intake of Sn from different food commodities was 66.8 µg, which was in accordance with the value of 1.12 µg/kg bw (per person weighted 60 kg). It was concluded that the main contribution to total Sn intake was made by vegetables, with substantial contributions from fruits, cheeses, and meats. A broader study indicated that the Sn intake from 100 g of cereal consumption was determined to be 1230 ng/day [61] and can be compared with the sample of flax seed flour. In contrast, the Sn intake from banana peel and grape seed flour exceeds this value. Above that, the Scientific Panel on Dietetic Products, Nutrition, and Allergies of the EFSA [33] concluded that the current total daily intake of Sn in the EU (ranging up to about 6 mg/day) appears to be well below the lowest intakes reported to cause metabolic symptoms; therefore, it is insufficient to derive a tolerable upper intake value for Sn. The previous data provided by EFSA even show a daily intake of Sn from cereals, excluding bread, of 78 µg [33]. Nevertheless, the JECFA adjusted the PTWI of 14 mg/kg bw [35]. In this study, non-cereal flours contribute less than 0.1% of the PTWI for Sn for each selected weight category.

**Figure 4:**
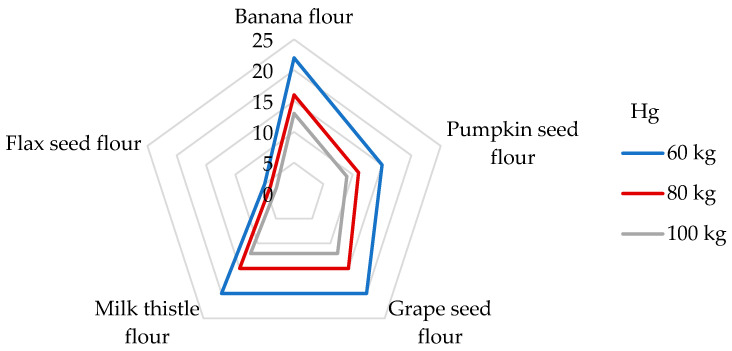
Dietary intake exposures (%) of Hg from non-cereal flours with respect to body weight (kg).

With regard to the presence of Hg in the flours tested in our study, the DI values were calculated for the individual non-cereal flours (Table 4). The highest DI value was unexpectedly found in banana peel flour (7.51 µg/day), whereas the lowest DI value was observed in flax seed flour (1.74 µg/day). Antoine [68] determined the daily intake of Hg from the consumption of a 100 g portion of rice up to 140 µg/g (corresponds to the DE_60_ 2.33 µg/kg bw), which is a much higher value than the DIs from non-cereal flours. The appropriate DE_60_ values of the non-cereal flours decrease in this order: banana peel (125 ng/kg bw) > grape seed > milk thistle > pumpkin > flax seed (29 ng/kg bw). A very low daily Hg intake of 1.13–1.48 μg was calculated from the 100 g portion of non-traditional wheat flakes [30]. If we compare our results with other research by consuming 100 g of maize grains, an estimated daily exposure of only 12.0 ng/kg bw was found [65]. The symptoms of Hg intoxication include cardiovascular, renal, neurological, and reproductive problems [68]. In the case of Hg, the risk characterization was performed by comparing the estimated exposure to the value of PTWI, which is established at 4 µg/kg bw [36,63]. The results of this study present the contributions of non-cereal fours to the PTWI of Hg as being from 5 to 22% for participants weighing 60 kg (Figure 4). Pumpkin, grape seed, milk thistle, and banana flours were found to be significant contributors to the PTWI value for Hg, whereas the contribution was greater than 15% [74]. In the study analyzing the toxic elements in wheat grains, a contribution value similar to the PTWI of about 4.3% was analyzed for flax seed flour [75]. Considering other studies, the DI value of the Hg intake from a 100 g portion of cornflakes was found to be 1.83 μg, corresponding to a contribution of 5% to the PTWI [51].

Estimated daily dietary intakes (DIs) and exposures (DE_bw_) by body weight values for Ni, As, Ag, and Pb from consumption of non-cereal flours are presented in Table 5.

**Figure 5:**
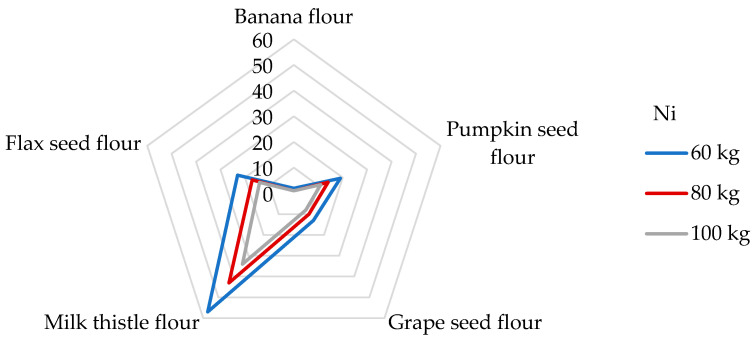
Dietary intake exposures (%) of Ni from non-cereal flours with respect to body weight (kg).

Ni, as a potentially toxic trace element, has been studied for its specific harmful responses in humans’ respiratory tracts and skin. It may serve as a cofactor of specific metallo-enzymes, for example, facilitating Fe absorption, hydrolysis, and redox reactions without the exception of gene expression [2,54,65,76]. In more detail, exposure to Ni affects the gastrointestinal and immune systems, hampers spermatogenesis, causes lung cancer and nasal sinus cancer, and may cause haematologic problems [52,65]. Taking into account these facts, Ni is ranked among compounds in the SPL [50]. Regarding food sources, cereal and cereal-based products, beverages, confectionery, and legumes were found to be the main contributors to Ni’s daily dietary intake value within different dietary sources [52]. Various Ni dietary exposure levels of 2.33 and 1.63 µg/kg bw have been reported in adults in France and the UK, respectively [2]. The Institute of Medicine (IOM) estimates a tolerable upper intake level (which means the highest level of daily intake that is likely to pose no risk of adverse health effects for almost all individuals) of 1 mg/day [76]; recently, the EFSA re-adjusted the tolerable daily intake (TDI) to 13 μg/kg bw [39]. Due to the lack of data, it has not been possible to establish PTWI or PTMI values that would be considered health protective; nevertheless, the TDI value is widely used by researchers to estimate the potential health risk. As Table 5 represents, a 100 g portion of the examined non-cereal flours respond to a daily Ni intake of from 13.4 to 443 μg/day, which, according to the IOM, proves the non-cereal flours to be safe. It appears that the most substantial contributor to the TDI level of Ni is milk thistle flour. The DE_60_ values of non-cereal flours were estimated in the range from 0.22 to 7.38 µg/kg bw. Given that, in the literature, no data are reported about the estimated daily intakes from non-cereal flours, it is challenging to compare results. Regarding similar research, the estimated daily Ni intakes from the consumption of 100 g portions of wheat and maize grains were 1040 and 520 µg/g, respectively [65]. The results obtained in this study are much higher than those found in non-cereal flours. In contrast, very low daily Ni intake values (2.20 and 1.93 µg/day, respectively) were obtained when eating 100 g of gluten-free flour and cereals [51,77]. Using the tolerable upper intake level suggested by IOM, we can establish that the contributions of non-cereal flours to this value are in a wide range of from 1 to 44% from one portion of non-cereal flours. When considering the TDI value set by the EFSA for Ni, the contributions of non-cereal flours were estimated to be between 2 and 57% for adults weighing 60 kg (Figure 5).

**Figure 6:**
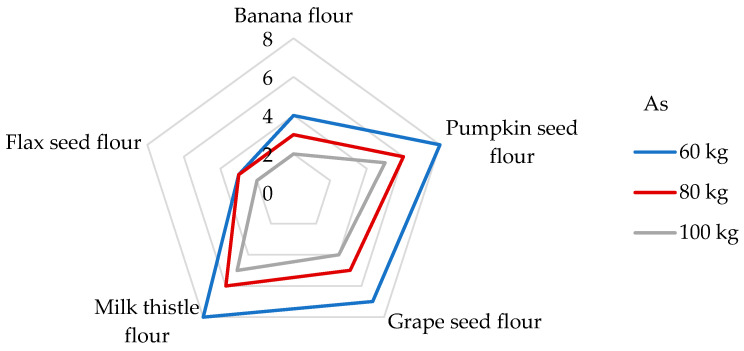
Dietary intake exposures (%) of As from non-cereal flours with respect to body weight (kg).

Long-term ingestion of As in humans may result in cardiovascular complications, neurotoxicity, skin lesions, cancer, developmental toxicity, abnormal glucose metabolism, and damaging molecules, such as DNA and proteins [56,65]. The IOM [76] indicates that As intake for all age groups ranged widely, from 0.50 to 0.81 µg/kg/day, and that the median intake was between 2.1 and 2.9 µg/day. The IOM also notes that there were not enough data to establish a tolerable upper intake level for As, and the data obtained clearly indicate the need for continued study to determine the metabolic role of As and to more fully characterize its specific functions in human health. Consequently, JECFA determined a PTWI value of 15 µg/kg/week (2.14 µg/kg/day). However, after further analysis, this value was withdrawn due to it being insufficiently protective and it has not been replaced to this day [72]. Additionally, the EFSA [78] reported a mean dietary exposure to inorganic As among adults ranging from 0.09 to 0.38 μg/kg bw per day, and, based on epidemiological studies, the JECFA identified a Benchmark dose lower confidence limit for a 0.5% increased incidence of lung cancer (BMDL0.5) of 3.0 μg/kg bw per day. To date, the TDI value of 0.3 µg/kg bw/day for As suggested by the World Health Organization (WHO) [40], and the same value presented as a reference dose for oral exposure (RfD) by the Environmental Protection Agency of the US (US EPA), are still applied in many studies [40,41,72,79]. Upon examination of non-cereal flours, the contribution of a 100 g portion to the TDI value of As is between 4 and 8% (Figure 6) and the DE_60_ values are in the range of from 10.5 to 24 ng/kg bw (Table 5). It is evident that the DE_60_ values are below the intakes presented by the IOM [76] and EFSA [78]. Usually, the predominant dietary source of the inorganic form of As is processed grains (non-rice-based products), followed by rice, milk, dairy products, and drinking water [78]. Compared to different types of cereal grains, the DE_60_ values measured for non-cereal flours are 2−22 times lower when the consumption of 100 g of cereal grains corresponded to daily exposures of from 173 to 518 ng As per kg bw [65]. The DI values evaluated for the consumption of non-cereal flours were found to be from 632 to 1440 ng/day (Table 5). However, the results of another study showed that the DI value in the case of gluten-free flour consumption may even reach 83 µg/day [51].

Soluble Ag substances can pose health risks, penetrate the body, accumulate according to their chemical form and the degree of exposure, and, in excess or in the long term, cause argyria after ingestion, diarrhea, decreased blood pressure, respiratory problems, and stomach irritation [62,80,81]. The toxicity of Ag occurs mainly in the aqueous phase and depends on the concentration of active, free Ag^+^ ions. Despite the fact that Ag was recognized as not carcinogenic, it has been added to the SPL for toxic substances [50]. The JECFA has not yet prepared or re-evaluated any toxicological reference values. However, according to the ICH guideline Q3D (R1) on elemental impurities, the established oral permitted daily intake (PDI) for Ag is 167 μg/day [81,82], which corresponds to dietary exposure of 2.78 μg/kg/day for a person weighing 60 kg. Regarding the daily consumption of 100 g of non-cereal flours, the estimated DI values were 23−661 ng/day (Table 5), and the appropriate DE_60_ values were in the range of from 0.38 to 11.0 ng/kg bw. This means that the consumption of non-cereal flours is safe. Additionally, the US EPA suggested an RfD of 5 µg of Ag per kg/bw/day, and this can still be applied [42]. The contributions of non-cereal flours to the RfD values are all below 0.1% in the mentioned weight categories of consumers. The scientific literature lacks articles on daily Ag intakes from banana, pumpkin, grape seed, milk thistle, and flax seed flours. Despite that, a portion of 100 g of rice may provide a maximum dietary intake of 130 µg/day [68]; the same portion of wheat flakes provides 507 ng/day [30].

The biological effect of Pb is associated with a wide range of health issues. Pb attacks all organ systems (neurological, behavioral, renal, etc.), increases mortality (mainly due to cardiovascular diseases) and hypertension, impairs fertility, and negatively affects cognitive development in children. It was recognized as a possible carcinogen [44,65,66,83,84]. Neither the US EPA nor EFSA have yet established the RfD or PTWI values for Pb; the previously published PTWI value for Pb (25 μg/kg bw/w) by the EFSA was withdrawn in 2002 [43,49]. To characterize the health risks of exposure to Pb, the EFSA prescribed applying the margin of exposure (MoE) approach that includes BMDL values for neurotoxicity (0.5 μg/kg bw/day), nephrotoxicity (0.63 μg/kg bw/day), and cardiovascular effects (1.5 μg/kg bw/day) [43]. Concerning the DI values for Pb intake, the consumption of 100 g of non-cereal flours may possess between 3.50 and 8.34 µg of Pb per day (Table 5). When comparing the data, a 100 g portion of wheat grains provides a daily intake of Pb of 8.80 µg [59], while wheat flakes were associated with a daily intake of about 3.40–5.80 µg [30]. On the other hand, the intake of Pb from the consumption of wheat grains may even reach 37.9 µg/day [75]. As the main food categories contributing to the daily intake of Pb for adults, cereal products (especially bread and rolls), beverages (beer, tea, etc.), tap water, vegetables, and vegetable products were recognized [43]. Regarding the DE_60_ values for Pb (Table 5), the dietary intake exposures were in the following order: flax seed flour (56.3 ng/kg bw) < milk thistle flour < banana flour < pumpkin seed flour < grape seed flour (139 ng/kg bw). The average exposure to Pb in the diet for adults in the EU ranged from 0.36 to 1.24 μg/kg bw concerning countries, while the highest exposure was found for adults from Chile (3 µg/kg bw/day) [85]. It could be emphasized that the exposure values to Pb intake from non-cereal flours are very low. Based on the EFSA opinion [43] on Pb, the dietary exposure assessment was performed to detect potential health risks. The MoE values for developmental neurotoxicity, nephrotoxicity, and cardiovascular effects obtained for current exposures to dietary intake of Pb were more significant than 1, which is considered a safety margin without health concerns (Table 6). There are no similar studies to assess the health risks associated with the consumption of non-cereal flours analyzed in our study using BMDL limits for Pb. To illustrate this point, the MoE values of non-cereal flours are comparable with the MoE values calculated in the study evaluating health risks associated with the consumption of daily meals originating in Cyprus [44].

### 3.3. Dry Matter, Ash Content, and Dry Matter Digestibility Assessment of Non-Cereal Flours

Table 7 revealed the proximate dry matter and ash content of banana, pumpkin, grape, milk thistle, and flax flours. The flour’s dry matter and ash content varied widely from 90.5 to 96.3% and 3.42 to 9.76%, respectively. Our results can be compared with the study presented by [16], where the dry matter values of the pumpkin seed flour ranged between 92.2 and 94.5%, but the ash content was half as much (3.79−4.41%). No legislative requirements for dry matter or moisture content for non-cereal flours measured in our study have yet been established. It was postulated that the technological processing of pumpkin seeds significantly influenced the ash content in the final pumpkin seed flour. If we consider that the ash content is an indicator of the total mineral content in food, then thistle seed flour can contain ten times more ash, than wheat bread flour [25]. Furthermore, our sample contained three times more ash content than determined by Menasra [25] in their study of roasted milk thistle seed flours. Banana and grape seed flours have a relatively poor ash content compared to our other samples. The ash content in bananas is known to decrease with the fruit’s increasing maturity stage, approximately from 7.03 to 3.05% [46].

### 3.4. Effect of the Digestion on Toxic Trace Element Retention in Non-Cereal Flours

Because one of the objectives of this study was to evaluate the retention factor (RF, %) for individual toxic elements (see Figure 1), it was essential to measure not only the amounts of trace elements in native but also in undigested parts of non-cereal flours. The concentrations of toxic trace elements determined after the digestion simulation under in vitro conditions are shown in Table 8. The concentrations of individual toxic trace elements were the basis for the calculation of RF values according to Equation (4).

**Figure 7:**
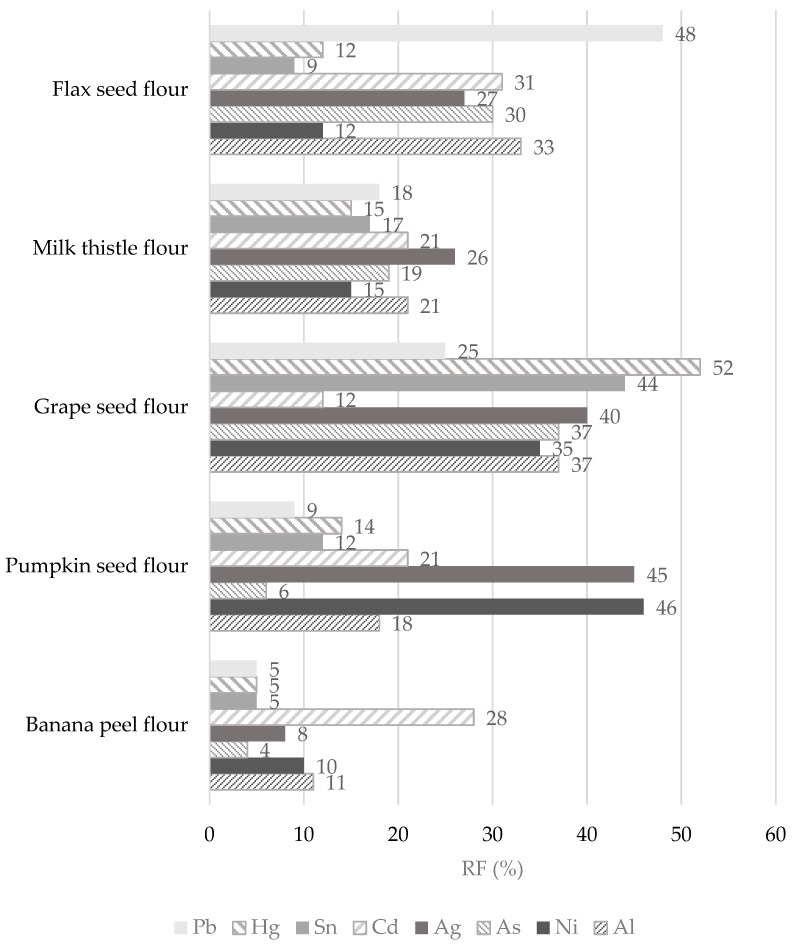
The remaining parts expressed as retention factor (RF, %) of toxic trace elements after simulation of in vitro digestion of non-cereal flours.

The contributions of non-cereal flours to the PTWI, PTMI, TDI, and RfD values for individual toxic trace elements calculated from their concentrations in samples are overestimated as the digestion processes may modify their bioaccessibility and retention from the matrix of the flours. To obtain the retention factor value (RF, %), the data presented in Table 3, Table 7, and Table 8 were applied using Equation (4). The results are presented in Figure 7. It can be seen that RF values for individual toxic trace elements are in wide ranges and that they seem to be dependent on the matrix of non-cereal flours in which the elements are bound in certain chemical forms. The lower the RF value, the lower the amount of toxic elements remaining in the matrix of the undigested part of the flour, so it is potentially more accessible for absorption in the gastrointestinal tract [30]. A comparison of the results with other studies is limited due to the scarce data provided under the same conditions for the digestion of non-cereal flours and the ability to obtain an undigested part of the flours for further analysis.

In the case of Pb, the results clearly show that it is easily released from banana and pumpkin flour matrices during the digestion process, while the highest RF value (up to 48%) is found in flax flour. In other words, while bioavailability is the part actually available and taken up by a human tract, bioaccessibility is an experimentally determined estimate of what is potentially bioavailable [86]. Thus, the RF value of 48% means that 52% of the Pb released from the flax seed flour matrix may be potentially absorbed (bioaccessible) by the human tract and 48% of Pb could pass into the large intestine. The digestion of Pb is influenced by physiological (age, nutritional calcium status, fasting) and physicochemical characteristics (solubility, Pb species), and the absorbed Pb values were in the range of from 15 to 20% for adults. In adults, the reported absorption of Pb when taken with a meal varied from 3 to 21%; when ingested by fasting subjects, the absorption of Pb was approximately 63% [84,86]. Hg has a high RF value only in the grape seed flour sample, namely, 52%, which is also the highest retention value measured among toxic elements. In contrast, the banana flour matrix recorded the lowest RF value for Hg (5%). The lowest RF values for banana flour were assessed for other elements, such as Pb, Sn, Ag, As, Ni, and Al. It should be highlighted that these toxic trace elements (except Cd) are easily released from the banana flour matrix and could probably be more easily bioaccessible into the small intestine. Some of the highest RF values for individual toxic trace elements were measured in the grape seed flour sample. Specifically, the RF values for toxic trace elements in the grape seed flour decreased in the following order: Hg (52%) > Sn (44%) > Ag (40%) > Al and As (37%) > Ni (35%) > Pb (25%), and Cd (12%) (Figure 7). It appears that 88% of Cd is released from the matrix of grape seed flour and may be absorbed by the human digestive tract. It could be stated that an undigested part of the grape seed flour remains with large amounts of toxic trace elements that can pass into the large intestine, followed by flax seed flour (RF values for Pb, Cd, Ag, As, and Al are above 20%) and pumpkin seed flour (RF values for Cd, Ag, and Ni reached above 20%). The bioaccessibility of Pb and Cd measured from consuming cereals, meats, and vegetables varies widely (5–80%). It depends on the food type, and the bioaccessibility values from the consumption of cocoa powder did not exceed 10 and 50% in the case of Pb and Cd, respectively [87]. However, despite the measured results from other studies, the EFSA states Cd bioavailability after dietary exposure in humans in relatively low values (3–5%) [70]. The bioaccessibility values for Al from animal foods are very low, up to 2.2% [87]. In contrast, in non-cereal flour samples, the RF values range from 11 to 37%, corresponding to bioaccessibility values of between 63 and 89%. Our results are comparable with those of the Sumczynski study [30], where the RF values of Al for the wheat flakes reached 35%. The bioavailability of Sn from the digestive tract in humans was recognized as very low, with as much as 98% being excreted directly in feces at intakes around 10 mg/day or higher [33]. Sn’s bioavailability and potential toxicity in food depend not only on the amount ingested but also on many other factors, such as pH, valence, extent of adsorption, and solubility [8]. The data presented in Figure 7 show that, during in vitro digestion, Sn is retained more in the grape seed flour matrix (RF 44%) and is most easily released from the banana flour matrix (RF 5%). During the digestion of non-cereal flours, Al is retained in their matrix from 11 to 37%, which corresponds to a bioaccessibility level of from 63 to 89% (Figure 7), whereas the subsequent bioavailability of Al from food is considered to be lower than 0.1% [32]. The situation is different for As, where inorganic forms are immediately entirely absorbed by the human digestive tract while organically bound forms are absorbed by 70% [58]. Regarding non-cereal flours, As is easily released from the matrix of banana and pumpkin flours and is least easily released from grape seed flour (Figure 7).

This part of the experiment shows that all toxic trace elements are still a part of the undigested portion of flours and, theoretically, could pass into the large intestine. What is more, it cannot be stated unequivocally that any of the elements would always have the lowest or highest RF value in each sample, but the sample matrix and digestion process itself will influence their retention. The retention of toxic elements, depending on their chemical form or the degree of oxidation in which they occur, could be demonstrated in the future by, for example, ion liquid chromatography connected to ICP-MS.

### 3.5. Effect of In Vitro Digestion on the Metal Pollution Index

Food and water are the main pathways through which humans are exposed to heavy metals [88]. In contrast to most contaminants, heavy metals are not biodegradable and accumulate in human body organs; therefore, food safety from the point of view of metal concentrations is of public concern worldwide [89]. Long-term consumption of heavy metal-contaminated foods may alter many metabolic processes and, subsequently, can lead to various kidney, liver, bone, and nerve disorders, in addition to impediments in immunological responses and numerous types of cancer [88,90]. Among heavy metals, some are toxic (such as Cd, Pb, Ag, Hg, As, Ni, Al, Sn, etc.), and some are essential (for example, Fe, Zn, Cu, Se, Cr, Mn, and Co) [30,89,90].

**Figure 8:**
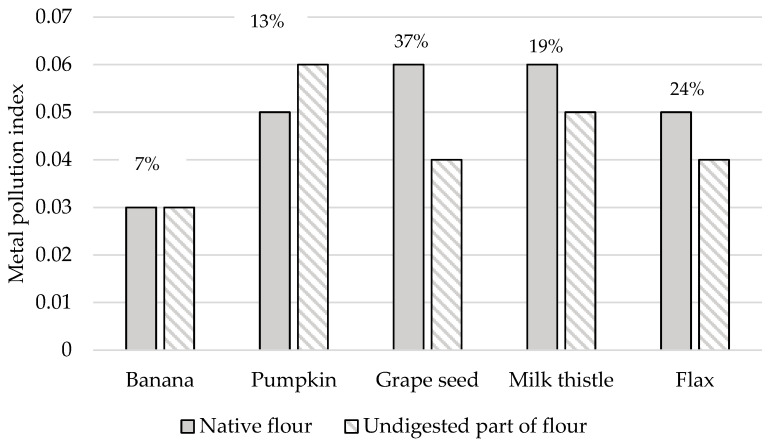
Metal pollution index of native and undigested parts of non-cereal flours. RP_MPI_—the remaining part of the metal pollution index after the digestion of the flours is expressed in % (above the columns for each sample).

This study computed the MPI value to determine the overall toxic heavy metal concentrations in different types of non-cereal flours analyzed (see Figure 8). In addition, Figure 8 also showed the RP_MPI_ values as percent to obtain the part of the MPI value that is still retained in the undigested part of the flours tested. Among the samples tested, the grape and milk thistle seed flours exhibited the highest MPI values of 0.06 for both samples, followed by the pumpkin and flax seed flours. The MPI values calculated in the case of the study of raw vegetables ranged from 1.62 to 11.2 [88]; in the case of fish, it was between 1.9 and 3.0 [89], and, for gluten-free bread, it was 0.44 [51]. These values were many times higher than our samples. Consequently, the results show that 7–37% of the MPI value assessed for native forms of flour remains in the undigested part of the samples, which agrees with the results reported in the study presented by Sumczynski [30].

## 4. Conclusions

This study provides data on the toxic trace element content in non-cereal flours and their undigested parts. In addition, it estimates dietary intakes and exposures to toxic elements for adults weighing 60, 80, and 100 kg and evaluates the metal pollution index. Furthermore, this study examines the retention factors of elements after the in vitro digestion process.

Al was found to be the most concentrated toxic element in non-cereal flours. All samples, except banana flour, had Ni concentrations two or more times higher than those measured in various foods consumed in the EU. The highest concentration of As was found in milk thistle and pumpkin seed flours, and the highest concentration of Ag was evaluated in the grape seed flour. Our study showed that flax seed and milk thistle flours contained the significantly highest Cd concentrations (247 and 132 ng/g, respectively), which were two orders of magnitude higher than those measured in other samples. The samples analyzed were found to have Hg and Pb contents in the order of tens of ng. In the case of consuming 100 g of banana flour, a consumer weighing 60 kg is most exposed to Al intake (12.0 µg/kg bw), followed by exposure to Ni, Hg, Pb, Sn, As, Ag, and Cd (1.77 ng/kg bw). When consuming pumpkin flour, the dietary exposures for an adult decrease in the following order: Al (13.8 µg/kg bw) > Ni > Pb > Hg > As > Cd > Sn > and Ag (3.07 ng/kg bw). Regarding grape seed flour, the dietary exposures decrease in the following order: Al (45.8 µg/kg bw) > Ni > Pb > Hg > Sn > As > Ag > and Cd (1.63 ng/kg bw). When consuming a 100 g serving of milk thistle seed flour, dietary exposure to toxic elements can be ranked in the following descending order: Al (11.9 µg/kg bw), Ni, Cd, Hg, Pb, As, Sn, and Ag (0.38 ng/kg bw). Finally, the dietary exposure values when consuming flax seed flour decrease in the following order: Al (8.70 µg/kg bw) > Ni > Cd > Pb > Hg > Sn > As > and Ag (0.88 ng/kg bw). The levels of dietary intake exposures of non-cereal flours for adults to the PTWI of Al ranged from 3 to 16%, while the highest contribution was found for the grape seed flour. The contributions of milk thistle and flax seed flours to the Cd PTMI values were significant, specifically, they were 26 and 49%, respectively. The results showed that the contributions of fours to the PTWI of Hg ranged from 5 to 22%, while all samples, instead of the flour of flax seeds, contributed significantly. In this research, samples contribute less than 0.1% of the PTWI for Sn and RfD for Ag in each weight category; similarly, the contribution of non-cereal flour to the TDI value for As was insignificant. When considering the TDI value for Ni, the contributions of milk thistle, flax seed, and pumpkin seed flours were estimated to be 19−57%. Finally, the margin values for developmental neurotoxicity, nephrotoxicity, and cardiovascular effects obtained for exposures to Pb intake were considered safe. The highest RF value (above 50%) for Hg in the grape seed flour was examined; all other RF values for toxic elements in all samples were below 50%. When the effect of digestion on the release of toxic trace elements was examined, all toxic elements were still a part of the undigested portion of the flours and, theoretically, could pass into the large intestine. Moreover, the results show that from 7 to 37% of the MPI value assessed for non-cereal flour remains in the undigested part of the samples.

In the future, more research is needed on the undigested part of the flour in terms of the toxic elements released that are still bound and their chemical form after digestion because of the possible carcinogenesis of the colon that they could induce. Furthermore, despite the fact that the maximum permitted concentration of Cd in milk thistle seed flour has not yet been determined, our measurements showed that this sample could be a source of Cd intake in the future. Considering the fact that the retention of toxic trace elements showed that they are retained most in the grape seed and pumpkin seed flours during the digestion process, these samples should be subjected to further analyses under in vivo conditions.

## Figures and Tables

**Table 1:** Specific working parameters.

**Power**	1550 W	**Cool gas flow rate**	14 L/min
**Sampling depth**	0.5 cm	**Auxiliary gas flow rate**	0.8 L/min
**Nebulizer gas flow rate**	1.015 L/min	**Flow rate of He**	4.1 mL/min
**Nebulizer pump speed**	40.00 rpm	**Chamber temperature**	2.7 °C

**Table 2:** The resulting values of certified materials measured by ICP-MS.

Reference Materials	Elements	Analysed Values ^a^	Reference Values ^a,b^	Recovery (%)
NIST 568b Rice flour	Al	4.25 ± 0.14	4.21 ± 0.34	101.0
	As	0.290 ± 0.010	0.285 ± 0.014	102.0
	Cd	0.0220 ± 0.0005	0.0224 ± 0.0013	98.2
	Hg	0.00582 ± 0.00027	0.00591 ± 0.00036	98.5
	Sn	0.0051 ± 0.001	0.0050 ± 0.0010	102.0
	Pb	0.0081 ± 0.0020	0.0080 ± 0.0030	101.3
Lichen (IAEA-336)	As	0.64 ± 0.04	0.63 ± 0.08	101.6
	Hg	0.19 ± 0.02	0.20 ± 0.04	95.0

^a^ Mean (mean value of five measurements) ± standard deviation. ^b^ Confidence interval 95%.

**Table 3:** Toxic trace elements determined in native non-cereal flours using ICP-MS.

Analyte (ng/g)	Banana Peel Flour	Pumpkin Seed Flour	Grape Seed Flour	Milk Thistle Flour	Flax Seed Flour
^27^Al	7180 ± 30 ^a^	8280 ± 40 ^b^	27500 ± 100 ^c^	7130 ± 30 ^d^	5220 ± 40 ^e^
^60^Ni	134 ± 10 ^a^	1480 ± 20 ^b^	990 ± 30 ^c^	4430 ± 30 ^d^	1810 ± 20 ^e^
^75^As	6.32 ± 0.22 ^a^	13.5 ± 0.3 ^b^	12.7 ± 0.3 ^c^	14.4 ± 0.2 ^d^	6.41 ± 0.12 ^a^
^107^Ag	1.50 ± 0.05 ^a^	1.84 ± 0.04 ^b^	6.61 ± 0.12 ^c^	0.23 ± 0.02 ^d^	0.53 ± 0.03 ^e^
^111^Cd	1.06 ± 0.02 ^a^	9.15 ± 0.10 ^b^	0.98 ± 0.04 ^c^	132 ± 8 ^d^	247 ± 5 ^e^
^118^Sn	14.7 ± 0.5 ^a^	5.07 ± 0.03 ^b^	14.6 ± 0.2 ^a^	9.58 ± 0.11 ^c^	11.4 ± 0.4 ^d^
^202^Hg	75.1 ± 0.5 ^a^	52.0 ± 1.5 ^b^	69.3 ± 0.6 ^c^	67.0 ± 0.9 ^d^	17.4 ± 0.2 ^e^
^208^Pb	37.6 ± 0.4 ^a^	53.5 ± 2.3 ^b^	83.4 ± 1.4 ^c^	35.0 ± 2.0 ^d^	33.8 ± 1.8 ^d^

All results are presented in dry matter as means ± SD, *n* = 9 (the mean of nine measurements). Means within a line with at least one identical lowercase superscript letter do not differ significantly (*p* ≥ 0.05).

**Table 4:** Estimated daily dietary intakes (DIs) and exposures (DE_bw_) from consumption of non-cereal flours.

Analyte	Banana Peel Flour	Pumpkin Seed Flour	Grape Seed Flour	Milk Thistle Flour	Flax Seed Flour
^27^Al					
DI (µg/day)	718	828	2750	713	522
DE_60_ (µg/kg bw)	12.0	13.8	45.8	11.9	8.70
DE_80_ (µg/kg bw)	9.00	10.4	34.4	8.91	6.53
DE_100_ (µg/kg bw)	7.18	8.28	27.5	7.13	5.22
^111^Cd					
DI (ng/day)	106	915	98	13,200	24,700
DE_60_ (ng/kg bw)	1.77	15.3	1.63	220	412
DE_80_ (ng/kg bw)	1.33	11.4	1.23	165	309
DE_100_ (ng/kg bw)	1.06	9.15	0.98	132	247
^118^Sn (ng/g)					
DI (ng/day)	1470	507	1460	958	1140
DE_60_ (ng/kg bw)	24.5	8.45	24.3	16.0	19.0
DE_80_ (ng/kg bw)	18.4	6.34	18.3	12.0	14.3
DE_100_ (ng/kg bw)	14.7	5.07	14.6	9.58	11.4
^202^Hg					
DI (µg/day)	7.51	5.20	6.93	6.70	1.74
DE_60_ (ng/kg bw)	125	86.7	116	112	29.0
DE_80_ (ng/kg bw)	93.9	65.0	86.6	83.8	21.8
DE_100_ (ng/kg bw)	75.1	52.0	69.3	67.0	17.4

**Table 5:** Estimated daily dietary intakes (DIs) and exposures (DE_bw_) from consumption of non-cereal flours.

Analyte	Banana Peel Flour	Pumpkin Seed Flour	Grape Seed Flour	Milk Thistle Flour	Flax Seed Flour
^60^Ni					
DI (µg/day)	13.4	148	99	443	181
DE_60_ (µg/kg)	0.22	2.47	1.65	7.38	3.02
DE_80_ (µg/kg)	0.17	1.85	1.24	5.54	2.26
DE_100_ (µg/kg)	0.13	1.48	0.99	4.43	1.81
^75^As					
DI (ng/day)	632	1350	1270	1440	641
DE_60_ (ng/kg)	10.5	22.5	21.2	24.0	10.7
DE_80_ (ng/kg)	7.90	16.9	15.9	18.0	8.02
DE_100_ (ng/kg)	6.32	13.5	12.7	14.4	6.41
^107^Ag					
DI (ng/day)	150	184	661	23	53
DE_60_ (ng/kg)	2.50	3.07	11.0	0.38	0.88
DE_80_ (ng/kg)	1.88	2.30	8.26	0.29	0.66
DE_100_ (ng/kg)	1.50	1.84	6.61	0.23	0.53
^208^Pb					
DI (ng/day)	3760	5350	8340	3500	3380
DE_60_ (ng/kg)	62.7	89.2	139	58.3	56.3
DE_80_ (ng/kg)	47.0	66.9	104	43.8	42.3
DE_100_ (ng/kg)	37.6	53.5	83.4	35.0	33.8

**Table 6:** Margins of exposure values (MoE) for Pb.

Population Class (bw)	MoE^1^	MoE^2^	MoE^3^	MoE^1^	MoE^2^	MoE^3^
	Banana flour			Pumpkin seed flour		
60 kg	8	10	24	6	7	17
80 kg	11	14	32	7	9	22
100 kg	13	17	40	9	12	28
	Grape seed flour			Milk thistle flour		
60 kg	4	5	11	9	11	26
80 kg	5	6	14	11	14	34
100 kg	6	8	18	13	18	43
	Flax seed flour					
60 kg	9	11	27			
80 kg	12	15	35			
100 kg	15	17	44			

MoE—margin of exposure, MoE^1^—developmental neurotoxicity (BMDL of 0.5 μg/kg bw/day), MoE^2^—nephrotoxicity (BMDL of 0.63 μg/kg bw/day), and MoE^3^—cardiovascular diseases (BMDL of 1.5 μg/kg bw/day) [43].

**Table 7:** Dry matter and ash contents and in vitro digestibility values of non-cereal flours.

Flours	Dry Matter (%)	* Ash (%)	Digestibility (%)
Banana peel flour	96.3 ± 0.2 ^a^	3.62 ± 0.03 ^a^	92.6 ± 0.2 ^a^
Pumpkin seed flour	91.7 ± 0.1 ^b^	8.84 ± 0.07 ^b^	89.1 ± 0.3 ^b^
Grape seed flour	90.5 ± 0.1 ^c^	3.42 ± 0.01 ^c^	44.0 ± 0.8 ^c^
Milk thistle flour	91.2 ± 0.1 ^d^	9.76 ± 0.03 ^d^	76.7 ± 0.7 ^d^
Flax seed flour	92.6 ± 0.1 ^e^	5.92 ± 0.01 ^e^	69.6 ± 1.5 ^e^

Means of triplicate determinations ± SD (*n* = 3). Means within a column with at least one identical small superscript do not differ significantly (*p* ≥ 0.05). * Ash content is presented on dry matter basis.

**Table 8:** Toxic trace elements determined in undigested part of non-cereal flours using ICP-MS.

Analyte (ng/g)	Banana Peel Flour	Pumpkin Seed Flour	Grape Seed Flour	Milk Thistle Flour	Flax Seed Flour
^27^Al	11,100 ± 100 ^a^	13,500 ± 100 ^b^	18,300 ± 100 ^c^	6350 ± 30 ^d^	5610 ± 30 ^e^
^60^Ni	174 ± 6 ^a^	630 ± 10 ^b^	610 ± 20 ^b^	2860 ± 20 ^c^	700 ± 15 ^d^
^75^As	3.21 ± 0.12 ^a^	7.81 ± 0.11 ^b^	8.33 ± 0.13 ^c^	11.7 ± 0.3 ^d^	6.33 ± 0.10 ^e^
^107^Ag	1.66 ± 0.06 ^a^	7.55 ± 0.05 ^b^	4.68 ± 0.10 ^c^	0.26 ± 0.02 ^d^	0.47 ± 0.02 ^e^
^111^Cd	3.96 ± 0.12 ^a^	17.3 ± 0.1 ^b^	0.21 ± 0.02 ^c^	120 ± 6 ^d^	254 ± 5 ^e^
^118^Sn	10.5 ± 0.5 ^a^	5.68 ± 0.10 ^b^	11.5 ± 0.5 ^c^	6.88 ± 0.08 ^d^	3.36 ± 0.12 ^e^
^202^Hg	55.6 ± 0.6 ^a^	67.2 ± 1.2 ^b^	64.6 ± 0.6 ^c^	44.5 ± 1.5 ^d^	6.71 ± 0.11 ^e^
^208^Pb	26.9 ± 0.4 ^a^	42.8 ± 2.2 ^b^	37.0 ± 1.2 ^c^	26.4 ± 1.8 ^a^	53.1 ± 2.3 ^d^

All results are presented in dry matter as means ± SD, *n* = 9 (the mean of nine measurements). Means within a line with at least one identical lowercase superscript letter do not differ significantly (*p* ≥ 0.05).

## Data Availability

Data are contained within the article. Further inquiries can be directed to the corresponding author.
